# Decision-analytic modeling for health economic evaluation in child and adolescent psychiatry using inpatient equivalent treatment - design of a doctoral thesis

**DOI:** 10.1192/j.eurpsy.2022.1137

**Published:** 2022-09-01

**Authors:** K. Bieniek, A. Neumann, B. Buchberger

**Affiliations:** 1 LWL Marl- Sinsen, Md- Management, Dortmund, Germany; 2 Universität Duisburg-Essen, Health Care Management And Research, Essen, Germany

**Keywords:** hometreatment, mental disorder, inpatient equivalent treatment, children and adolescents

## Abstract

**Introduction:**

Despite the high prevalence of psychiatric disorders among children and adolescents, services are often accessed too late or not at all. Inpatient-equivalent treatment can be a good option here, as it can counteract structural barriers by enabling treatment from home. Although national and international studies highlight the benefits, this form of treatment is offered by only a few psychiatric facilities.

**Objectives:**

The aim is to provide a decision-making aid for the introduction of outreach treatments with regard to cost-effectiveness. Based on this, the question will be answered whether telemedicine can be an option for the distribution of rare (human) resources.

**Methods:**

1) Based on a systematic review, the best available evidence will serve for deriving hypotheses and providing assumptions for the decision-making model. 2) Decision analytic modeling will be used to determine the cost-effectiveness of inpatient-equivalent treatment compared to conservative inpatient treatment. 3) An additional systematic review will provide information on the use of telemedicine in inpatient equivalent treatment.

**Results:**

The following questions need to be discussed: 1)Is there an indication for all psychiatric diseases and age groups? 2) Are there ethical considerations that need to be taken into account, especially in the use of telemedicine? What incentives need to be set for psychiatrists to opt for inpatient-equivalent treatment?

**Conclusions:**

The results of the study may help to raise awareness of inpatient equivalent treatment among decision-makers. Furthermore, fears could be reduced, since admission to a psychiatric facility can mean a stigmatizing intervention in the lives of young patients.

**Disclosure:**

No significant relationships.

